# Predicting corneal refractive power changes after orthokeratology

**DOI:** 10.1038/s41598-021-96213-x

**Published:** 2021-08-17

**Authors:** Pauline Kang, Vinod Maseedupally, Paul Gifford, Helen Swarbrick

**Affiliations:** School of Optometry and Vision Science, UNSW, Sydney, Australia

**Keywords:** Refractive errors, Vision disorders

## Abstract

This study aimed to characterise corneal refractive power (CRP) changes along the principal corneal meridians during orthokeratology (OK). Nineteen myopes (mean age 28 ± 7 years) were fitted with OK lenses in both eyes. Corneal topography was captured before and after 14 nights of OK lens wear. CRP was calculated for the central 8 mm cornea along the horizontal and vertical meridians. The central-paracentral (CPC) power ratio was calculated as the ratio between maximum central and paracentral CRP change from individual data. There was a significant reduction in CRP at all locations in the central 4 mm of the cornea (all p < 0.001) except at 2 mm on the superior cornea (p = 0.071). A significant increase in CRP was evident in the paracentral zone at 2.5, 3 and 3.5 mm on the nasal and superior cornea and at 3.5 and 4 mm on the temporal cornea (all p < 0.05). No significant change in CRP was measured in the inferior cornea except decreased CRP at 2.5 mm (p < 0.001). CPC power ratio in the nasal and temporal paracentral regions was 2.49 and 2.23, respectively, and 2.09 for both the inferior and superior paracentral corneal regions. Our results demonstrates that OK induced significant changes in CRP along the horizontal and vertical corneal meridians. If peripheral defocus changes are inferred from corneal topography, this study suggests that the amount of myopia experienced on the peripheral retina was greater than twice the amount of central corneal power reduction achieved after OK. However, this relationship may be dependent on lens design and vary with pupil size. CPC power ratios may provide an alternative method to estimate peripheral defocus experienced after OK.

## Introduction

Orthokeratology (OK) lenses are increasingly used for myopia control following recent studies which have consistently demonstrated effective myopia control in children fitted with OK compared to conventional single vision corrections^1–6^. Changes in corneal topography after overnight OK are believed to induce a hyperopic shift on to the central retina and myopic defocus on to the peripheral retina of myopes who typically experience peripheral hyperopic defocus with conventional corrections^7–12^. Recent animal studies have revealed that visual signals deriving from the peripheral retina have a profound impact on ocular growth; peripheral myopic defocus has an inhibitory effect on ocular growth while the opposite effect is evident with peripheral hyperopic defocus^13–16^. Thus it has been hypothesized that inducing myopic defocus on to the peripheral retina of progressive myopes may slow down or stop the progression of central myopia^16–18^, and that the peripheral myopic defocus induced by OK may be responsible for the reported myopia control effects with this modality^17,19,20^.

One of the main limitations of studies exploring peripheral optical defocus changes induced by OK is the sensitivity of instrumentation. The Shin-Nippon NVision-K 5001 autorefractor is currently the most commonly used instrument to measure optical profiles in myopia research^21–25^, however a limitation is that it utilizes three arcs of infrared light arranged in a 2.3 mm diameter circle rather than a single measurement point to derive refraction measurements^26^. Thus subtle changes in refraction may be masked by the large sampling area when using the autorefractor. An alternative and potentially superior method to describe and infer changes in peripheral optical defocus after OK is to characterize changes in corneal topography. Although changes in corneal topography may not be wholly analogous to changes in peripheral refraction, this approach may provide more detailed insight into optical changes induced by OK. Previous studies have described changes in corneal topography after OK^27–29^, but no studies to date have systematically correlated and determined relationships between corneal topography and refraction changes to provide a more direct method of estimating the optical change experienced after OK. This may be valuable to clinicians who do not have a means of measuring peripheral refraction.

A previous study which utilized autorefraction investigated the relationship between baseline spherical refractive error and induced peripheral myopic shift from OK to calculate an almost 1:1 relationship between baseline spherical equivalent central refractive error and induction of myopic refractive shift at 30° in the nasal and temporal visual fields^30^. This relationship, which is often used to infer peripheral refraction changes induced by OK, is not without limitations. More specifically, corneal topography and peripheral refraction changes vary significantly between individuals and are highly dependent on the OK lens fit. Thus, presenting mean data may not accurately reflect the peripheral defocus experienced by the individual.

The aim of the current study was to gain greater understanding of the optical changes induced by OK by comprehensively describing changes in corneal refractive power (CRP) across the horizontal and vertical corneal meridians, and from this to ascertain relationships between the amount of central corneal flattening and mid-peripheral corneal steepening in terms of CRP change.

## Materials and methods

### Subjects

A total of 19 young adult myopic subjects were enrolled (6 M, 13 F; mean age 28 ± 7 years). All subjects were required to have good ocular and general health, no previous rigid gas-permeable lens wear, and soft contact lens wearers were instructed to cease lens wear at least 24 h prior to study commencement. Central refraction was between − 1.00 and − 4.00 D with ≤ 1.50 D of astigmatism.

All subjects gave written informed consent to study participation after the nature of the study and risks and benefits of study participation were disclosed.

### Study design

This study was embedded in a larger study exploring the effects of OK on peripheral refraction along the vertical and horizontal meridians, details of which have been described previously^31^. In brief, subjects were fitted with BE OK lenses (Capricornia Contact Lens, Australia) in both eyes made from Boston XO_2_ material (Dk ISO/Fatt 141). BE OK lenses have an optic zone diameter of 6 mm and a total lens diameter of 11 mm. Study visits were scheduled at baseline before lens wear, and after 14 nights of OK treatment. Measurements from the right eye only are reported.

The research conducted in this study conformed to the tenets of the Declaration of Helsinki and the study received approval from the University of New South Wales Human Research Ethics Committee prior to study commencement. Further, all measurements were carried out in accordance with relevant guidelines and regulations.

## Measurement techniques

### Objective refraction

Non-cycloplegic central refraction measurements were taken using the Shin-Nippon NVision-K 5001 autorefractor (Tokyo, Japan). Conventional sphero-cylindrical refraction, converted into power vectors M, J_180_ and J_45_ using the equations derived by Thibos et al.^32^, are reported.

### Corneal topography

The Medmont E300 videokeratoscope (Medmont Pty Ltd, Melbourne, Australia) was used to capture corneal topography images and data were analyzed using Medmont Studio 6. Raw corneal topographic data including radial distance, sagittal height, axial curvature, tangential curvature and slope files were imported into a customized MATLAB (The MathWorks, Inc, Version 7.10) program. Each of these files contained matrices of up to 300 × 32 data points. Each row in the matrix represented a corneal hemi-meridian centred to the vertex normal or videokeratoscopic center and the hemi-meridians were separated by 1.2°. The MATLAB program interpolated data along each hemi-meridian at fixed intervals of 0.50 mm. CRP was then determined at each position along all meridians using the following formula described by Klein and Mandell^33^:

$$P_{R} = \frac{{n^{\prime}}}{{z + \frac{x}{{{\text{tan}}\left( {\theta_{i} - \theta_{t} } \right)}}}},$$

where *n*′ is the keratometric refractive index (1.3375), *z* is the sagittal height (mm), *x* is the radial distance (mm) from the videokeratoscopic center, *θ*_i_ is the incident ray angle and *θ*_t_ is the refracted ray angle. *θ*_i_ and *θ*_t_ were determined using the following equations^34^:

$$\theta_{i} = {\text{sin}}^{ - 1} \left( {\frac{x}{{r_{a} }}} \right),$$

$$\sin \theta_{t} = \frac{{\sin \theta_{i} }}{{n^{\prime}}},$$

where *r*_*a*_ is the axial radius of curvature (mm) determined by the corneal topographer at radial distance *x* mm from the vertex normal*.*

**Figure 1:**
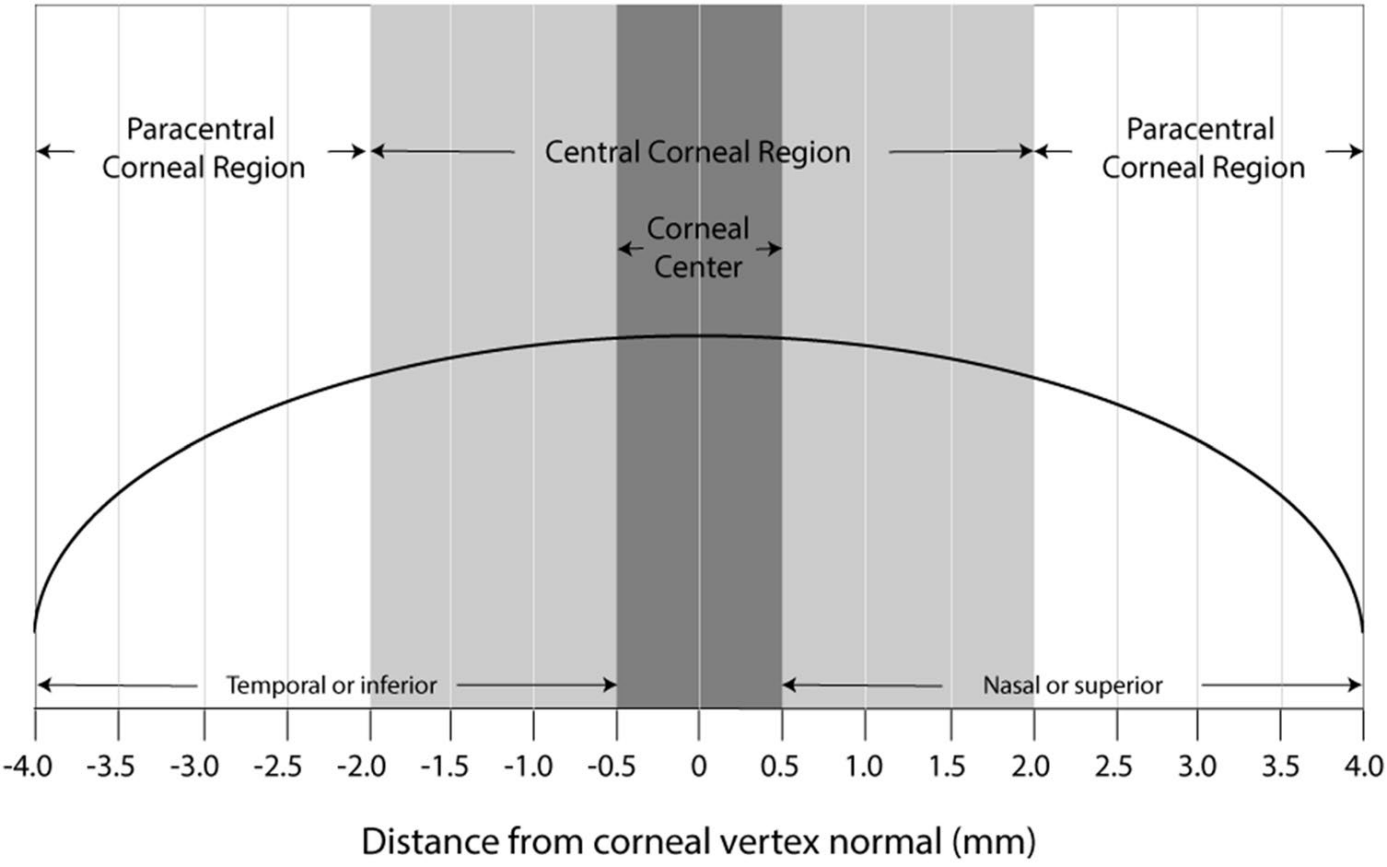
Diagram demarcating corneal center, and central and paracentral corneal regions.

CRP data were then averaged across four maps at each visit. Pre and post-treatment data along the horizontal and vertical meridians were retrieved at 0.50 mm interval steps to analyze change from baseline in CRP after OK. Data across the horizontal and vertical corneal meridians were divided into the corneal center, and central and paracentral regions; the central 1 mm corneal chord was defined as the corneal center (2 corneal locations) while the central 4 mm corneal chord was considered as the central corneal region (8 corneal locations), and the adjacent 2.5–4 mm areas (5–8 mm corneal chord) as the paracentral corneal regions (4 corneal locations in each paracentral region, 8 locations in total along each corneal meridian), as illustrated in Fig. 1.

### Data analysis

A Shapiro–Wilk test was used to test parametric distribution of data sets. Depending on normality, either paired t-test or Wilcoxon signed-rank test was used to assess changes in central refraction and corneal topography parameters after OK lens wear.

Linear mixed model analysis was used to detect overall changes in CRP along the horizontal and vertical corneal meridians after OK lens wear. If significant, t-tests with Bonferroni correction were used to identify areas of significant CRP change, and to detect asymmetry in the CRP change along each corneal meridian. A critical p value of 0.05 was used to denote statistical significance.

### Central-paracentral (CPC) power ratio

To estimate the amount of peripheral myopic shift experienced after OK for each subject, the ratio between the CRP change at the corneal center and the maximum CRP change in the paracentral region was individually calculated and termed the central-paracentral or CPC power ratio. The maximum value was selected rather than averaging CRP across the paracentral region as averaging values carries the risk of masking significant paracentral corneal changes, particularly if the treatment zone is decentered or larger than the central region. A correlation analysis was also conducted to determine any relationship between CRP changes at the corneal center and paracentral corneal region.

## Results

### Central refraction and topography

Central refraction and corneal topography parameters at baseline and after 2 weeks of OK lens wear are shown in Table 1.

**Table 1:** Objective central refraction M, J_180_ and J_45_ (D), apical radius (r_0_; mm), and Flat K and Steep K (D) values at baseline (BL) and after OK lens wear.

	BL	OK	p-value
M (D)	− 1.54 ± 0.77	+ 0.23 ± 0.63	0.020
J_180_ (D)	− 0.11 ± 0.17	− 0.18 ± 0.22	0.157
J_45_ (D)	0.00 ± 0.12	+ 0.03 ± 0.19	0.501
r_o_ (mm)	7.80 ± 0.22	8.18 ± 0.24	< 0.001
Flat K (D)	43.10 ± 1.18	41.53 ± 0.96	< 0.001
Steep K (D)	43.78 ± 1.22	42.37 ± 1.01	< 0.001

A significant hyperopic M shift was evident after 14 days of OK lens wear, with no change in astigmatism components. Furthermore, there was significant central corneal flattening indicated by significant decrease in Flat and Steep K values, and increase in apical radius of curvature (Table 1).

### Mean corneal refractive power change

**Figure 2:**
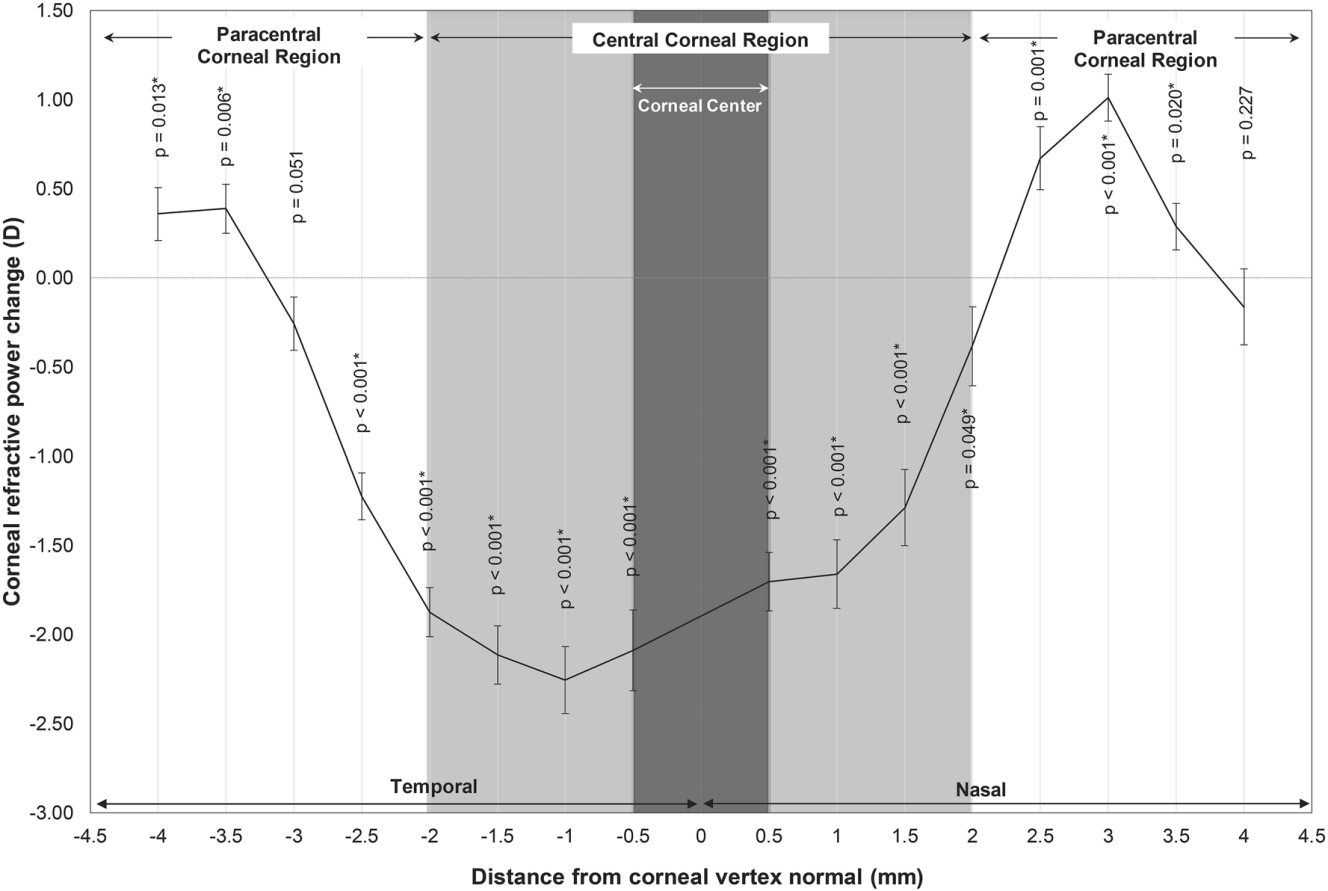
Mean corneal refractive power change after 14 nights of BE OK lens wear along the horizontal corneal meridian. Error bars represent standard error of the mean. Positive distance values denote the nasal cornea and negative values denote the temporal cornea. Asterisks indicate location of significant change.

After 14 nights of OK lens wear, along the horizontal corneal meridian, CRP significantly changed from baseline in the central corneal region (F_1, 270_ = 833.69, p < 0.001). Pairwise comparisons indicated significant reduction in mean CRP from baseline at all locations of the central region (all p < 0.001; Fig. 2). There was also a significant change in mean CRP in the paracentral region after OK (F_1, 288_ = 5.97, p = 0.015) with a significant increase in mean CRP at all paracentral locations on the nasal cornea except at 4 mm (− 0.16 ± 0.93 D, p = 0.227; Fig. 2). On the temporal cornea, a significant reduction in mean CRP was noted at 2.5 mm (− 1.22 ± 0.57 D, p < 0.001), and an increase in mean CRP at 3.5 (0.39 ± 0.60 D, p = 0.006) and 4 mm (0.36 ± 0.65 D, p = 0.013). Asymmetry in mean CRP change after OK was evident between the nasal and temporal cornea; there was a greater increase in mean CRP in the nasal than in the temporal paracentral cornea (p < 0.001; Fig. 2).

**Figure 3:**
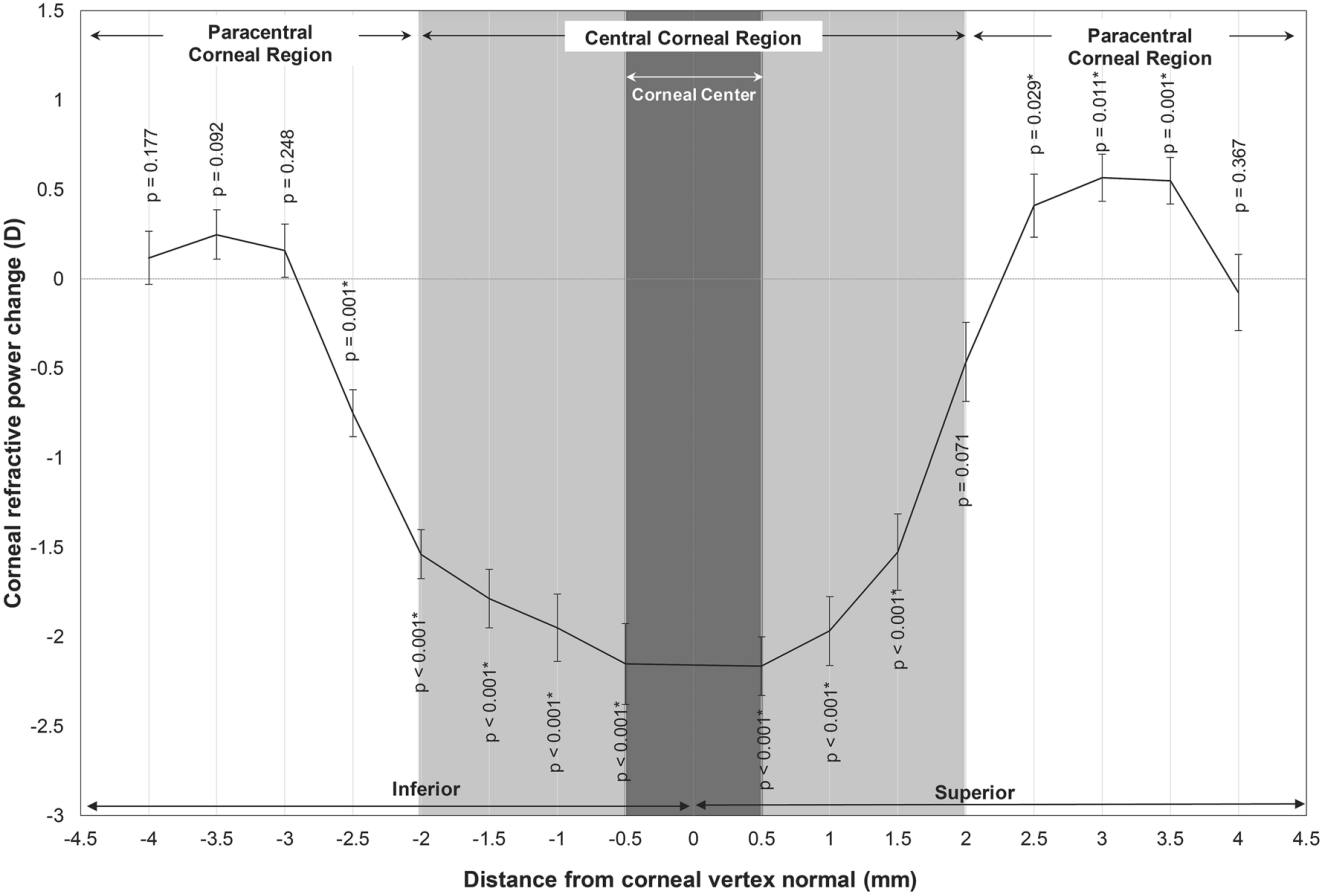
Mean corneal refractive power change after 14 nights of BE OK lens wear along the vertical corneal meridian. Error bars represent standard error of the mean. Positive distance values denote the superior cornea and negative values denote the inferior cornea. Asterisks indicate locations of significant change.

Along the vertical meridian, OK lens wear induced significant changes in mean CRP in the central region (F_1,270_ = 449.98, p < 0.001). There were significant reductions in mean CRP from baseline at all locations in the central region except at 2 mm on the superior cornea (− 0.46 ± 1.31 D, p = 0.071; Fig. 3). There was also a significant change in mean CRP in the paracentral zone (F_1,257.59_ = 4.62, p = 0.032). Pairwise comparisons revealed a significant increase in mean CRP at all locations (all p < 0.001) on the superior cornea except at 4 mm (− 0.07 ± 0.72 D, p = 0.367). On the inferior cornea, significant reduction in mean CRP was noted at 2.5 mm (− 0.75 ± 0.94 D, p = 0.001; Fig. 3) and increases in mean CRP in more inferior locations failed to reach statistical significance. Furthermore, there was no significant difference in mean CRP change in the superior compared to inferior paracentral corneal region (p = 0.063).

### CPC power ratio

Maximum CRP change was calculated individually for all subjects relative to the corneal center. Along the horizontal meridian, the mean CRP change at the corneal center was − 1.92 ± 0.78 D (range − 3.20 to − 0.75 D). Relative to the corneal center, there was a mean maximum CRP change of 4.72 ± 0.99 D (range 3.05 D to 7.39 D) in the nasal paracentral region and 4.24 ± 1.00 D (range 2.42 D to 5.87 D) in the temporal paracentral region. The CPC power ratio in the nasal and temporal paracentral regions relative to corneal center was 2.49 and 2.23, respectively.

Along the vertical meridian, the mean CRP change at the corneal center was − 2.16 ± 0.89 D (range − 3.81 D to − 0.84 D). In the superior paracentral region, the mean maximum CRP change relative to corneal center was 4.52 ± 1.30 D (range 2.20 D to 7.11 D). In the inferior paracentral region, the mean maximum CRP change relative to corneal center was 4.51 ± 1.36 D (range 2.26 D to 6.78 D). The CPC power ratio in the superior and inferior paracentral regions relative to corneal center was 2.09 and 2.09, respectively.

### Correlation analysis

To determine if there was a relationship between the amount of central corneal flattening and paracentral corneal steepening, Pearson correlation analysis was conducted on corneal center CRP change and paracentral region CRP change relative to corneal center along the horizontal and vertical meridians measured individually for each subject.

**Figure 4:**
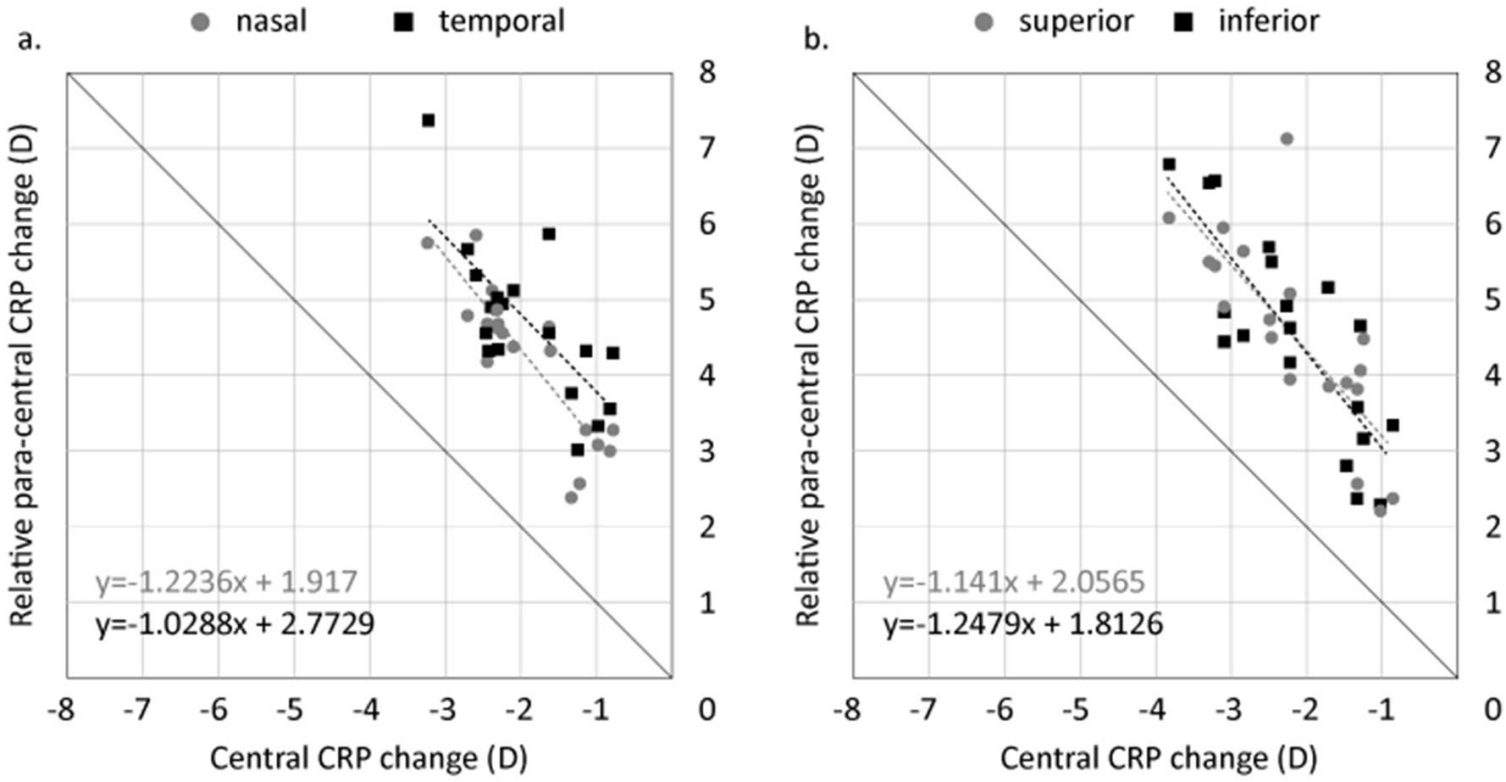
(**a**) Correlation between corneal center and relative paracentral CRP changes along the (**a**) horizontal and (**b**) vertical meridians. Bold line indicated 1:1 relationship. The dashed black line represents the relationship between the paracentral and corneal center CRP change for the temporal and inferior cornea, while the dashed gray line represents the relationship between the paracentral and corneal center CRP change for the nasal and superior cornea.

There was a significant correlation between corneal center CRP change and relative nasal (r = − 0.871; p < 0.001) and temporal (r = − 0.739; p < 0.001) CRP changes as shown in Fig. 4a. Similarly along the vertical meridian there was a significant correlation between corneal center CRP change and relative superior (r = − 0.781, p < 0.001) and inferior (r = − 0.819; p < 0.001) CRP changes (Fig. 4b).

## Discussion

There is a growing interest in OK following studies which have repeatedly demonstrated effective myopia control with OK compared to conventional corrections^1–6^. Animal studies have shown that peripheral retinal defocus has a significant impact on ocular growth^13–15^. Thus, peripheral myopic defocus induced by overnight OK is believed to be responsible, to some extent, for its reported myopia control effects. In the current study, we analyzed corneal topography changes as an alternative method to infer peripheral retinal defocus changes induced by OK. Significant central corneal flattening or decrease in CRP, and paracentral corneal steepening or increase in CRP was measured along the horizontal and vertical corneal meridians after 14 nights of OK compared to baseline. However, mean changes in CRP shown in Figs. 2 and 3 underestimate true individual differences in CRP between the central and paracentral corneal regions. Thus, we calculated CPC power ratio based on individual maximum changes in CRP to gain a better understanding of the optical changes induced by OK. CPC power ratio was calculated to be 2.49 and 2.23 for the nasal and temporal paracentral corneal regions and 2.09 for the superior and inferior paracentral regions. Thus, in terms of refractive power change, the maximum amount of relative paracentral corneal steepening that was achieved in this study with BE OK lenses was greater than twice the amount of central corneal flattening. Furthermore, there was a significant correlation between the amount of corneal center CRP change and relative paracentral CRP change along the horizontal and vertical corneal meridians as illustrated in Fig. 4.

Overall mean changes in central and paracentral CRP measured after 14 nights of OK lens wear are consistent with a previous study which measured significant increase in CRP in the nasal and superior paracentral corneal zones after OK^27^. Interestingly, Maseedupally et al.^27^ measured a reduction in CRP in the temporal paracentral corneal region. Discrepancy in results between the two studies may be due to differences in experimental protocols. Maseedupally et al.^27^ averaged CRP change across a sector in contrast to the current study where maximum changes in CRP were selected within the paracentral annular corneal regions along the horizontal and vertical meridians. Averaging across a corneal region may mask subtle changes, particularly on a cornea undergoing OK where there are areas of significant corneal flattening adjacent to areas of corneal steepening.

Asymmetry in CRP change was evident in the horizontal meridian, with greater increase in CRP in the nasal compared to temporal cornea as evident in Fig. 2, possibly due to treatment zone decentration. Treatment zone decentration has often been attributed to regional variations in corneal shape; the temporal cornea is often measured to be less prolate compared to the nasal cornea leading to temporal lens decentration^35–37^. Significant treatment zone decentration can lead to unfavourable visual outcomes due to induced astigmatism^38^ and higher order aberrations^39^. In contrast to the horizontal meridian, the vertical meridian does not exhibit asymmetry in corneal shape^37^ and accordingly, asymmetry in CRP change after OK was not evident along this meridian in the current study.

Kang and Swarbrick^31^ reported relative changes in spherical equivalent (M) peripheral refraction, in the same group of subjects as the current study, of − 1.80 and − 2.77 D at 35° in the nasal and temporal retinas, respectively, and − 2.75 and − 1.32 D at 30° in the inferior and superior retinas, respectively, after 14 days of OK. Mean changes in peripheral refraction were analysed using the same methodology adopted in Figs. 2 and 3, and not individualised maximum changes. Subsequent reanalysis of peripheral refraction data to calculate the maximum myopic shift in peripheral refraction for individual subjects indicated that after OK, along the horizonal meridian, a maximum of 1.90 and 2.78 D of myopia relative to the center in the nasal and temporal retinas, respectively, was induced. Similarly, along the vertical meridian, maximums of 2.76 and 1.32 D of myopia relative to the center in the inferior and superior retina, respectively, were measured. The change from baseline in central M after OK was + 1.77 D indicating that the amount of peripheral myopia induced on to the peripheral retina was less than twice the amount of achieved central refractive error correction. The inconsistencies between this reanalysis based on peripheral refraction data and the analysis reported in this paper based on corneal topographic data highlights the limitations of autorefraction in quantifying localised peripheral power changes.

Quieros et al.^30^ calculated an almost 1:1 relationship between baseline spherical equivalent refractive error and peripheral myopic defocus measured after OK, using peripheral refraction data. Based on this simple relationship, it is often mistakenly thought that the peripheral defocus experienced after OK is equivalent to the amount of central correction. Instead, this relationship implies a shift in peripheral myopic defocus relative to the central cornea that is approximately twice the amount of central correction, which is in agreement with the current study. By calculating CPC power ratios, the current study presents a more direct method to estimate the amount of peripheral myopic defocus experienced after OK rather than reporting changes in peripheral defocus relative to baseline^30^.

The individual locations of greatest increase in CRP in the paracentral regions were selected in this analysis rather than considering averaged data at set corneal locations in an attempt to accurately characterize individual peripheral defocus changes, and minimize the effects of variability in CRP change between subjects. For example, one subject had significant increase in CRP at 3 mm from the center along the vertical meridian while another subject demonstrated significant decrease in CRP at the same location. However, data were extracted along the vertical and horizontal corneal meridians only. To gain better understanding of corneal topography and hence peripheral refraction changes induced by OK, analysis of CRP change along other corneal meridians is required. Further, changes described here may be specific to the BE lens design and corneal changes may be different for lenses of other designs, particularly in relation to the reverse curve parameters and back optic zone diameter. Recent studies have also demonstrated reduced orthokeratology lens back optic diameters can decrease treatment zone diameters^40,41^.

Pupil size can also impact the optical defocus experienced on the peripheral retina^42^. Using ray tracing for different pupil sizes, Miguel et al. reported myopic shift in peripheral refraction profile when pupil diameters increased from 3 to 6 mm^42^. In the current study, pupil diameters were not standardized.

This study comprehensively characterized changes in CRP along the horizontal and vertical meridians after OK. CPC power ratios demonstrate that the amount of paracentral corneal steepening was at least twice the amount of corneal central flattening along the horizontal and vertical corneal meridians. If peripheral defocus changes are inferred from corneal topography, this indicates that the amount of peripheral myopic defocus experienced after OK is equivalent to twice the amount of central correction. However, this relationship may be dependent on lens design and vary with pupil size. CPC power ratios provide an alternative method to estimate the amount of peripheral myopic defocus experienced after OK.
